# Esophageal Perforation of a Neonate Following Placement of an Oral Gastric Tube

**DOI:** 10.7759/cureus.44461

**Published:** 2023-08-31

**Authors:** Trevor Borries, Luke W Eldore, John Burris, Zubin Shah, Kenneth Ford

**Affiliations:** 1 Diagnostic Radiology Residency, Baylor University Medical Center, Dallas, USA; 2 Medical School, Texas A&M College of Medicine, Dallas, USA; 3 Radiology, Baylor University Medical Center, Dallas, USA

**Keywords:** ph probe, esophagram, premature neonate, procedural complications, oral-gastric tube, neonatal intensive care unit (nicu), esophageal perforation

## Abstract

We present a case of neonatal esophageal perforation following routine oral gastric (OG) tube placement in the neonatal intensive care unit. This is a rare complication primarily affecting premature infants and can have significant morbidity and mortality. This case demonstrates the initial radiographic presentation of esophageal perforation and the subsequent imaging to confirm the diagnosis. Clinical management of this condition in the neonatal patient is also discussed. A unique highlight of this case is the difference in radiographic presentation on the initial study as compared to the later study.

## Introduction

Esophageal perforation in premature neonates is a rare and potentially devastating complication following enteric tube placement [1]. The incidence of esophageal perforation is approximately 0.006% (approximately six per 1,000 patients); however, it should be noted this statistic factors in all iatrogenic causes of esophageal perforation [2,3]. Mortality rates are estimated to be between 20% and 28% following perforation [4]. The complication rate could not be found well described in the current literature. The most commonly recognized risk factor for neonatal esophageal perforation is low birth weight due to the smaller and weaker muscular layer of the esophagus [5]. Other risk factors are not well recognized in the literature. Recognition of esophageal perforation by the clinical team and coordination with a radiologist can allow for prompt diagnosis and treatment.

## Case presentation

A 33-year-old woman (gravida 2, para 1, abortion 1) without prenatal care presented with acute abdominal pain. She was not given betamethasone and subsequently delivered a baby boy at 23 weeks and two days on October 3, 2022. A physical exam at birth showed bilateral and symmetrical chest expansion with bilateral coarse crackles and an otherwise normal exam for a premature infant of this gestational age. The patient's birth weight was 580 g, and his APGAR scores were 6 and 8 at one and five minutes, respectively. This patient was intubated, placed on positive pressure ventilation (PPV), and given surfactant in the delivery room. The intubation was not noted as difficult by the neonatal nurse practitioner who performed it. The patient was then moved to the neonatal intensive care unit (NICU), where other management, such as placing an oral gastric feeding tube (OGT) with an X-ray confirming placement, was begun.

**Figure 1:**
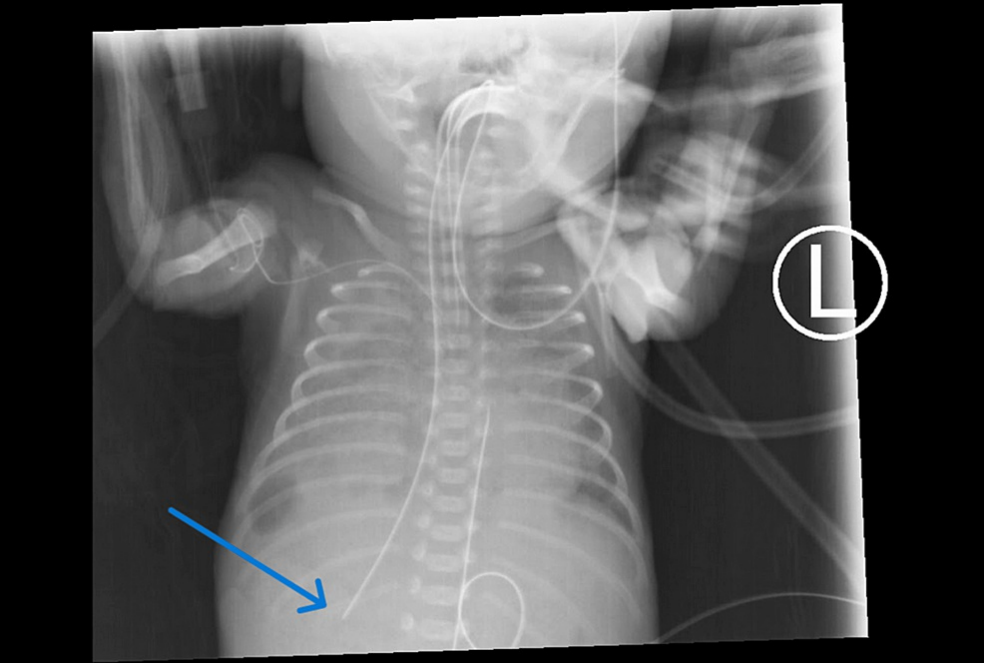
Abnormal oral gastric tube positioning on a chest radiograph. The tube can be visualized as coursing down on the right side of the patient in an inferolateral fashion with termination in the area of the right lung base. This could possibly indicate abnormal anatomy or, as in this case, abnormal positioning of the oral gastric tube.

**Figure 2:**
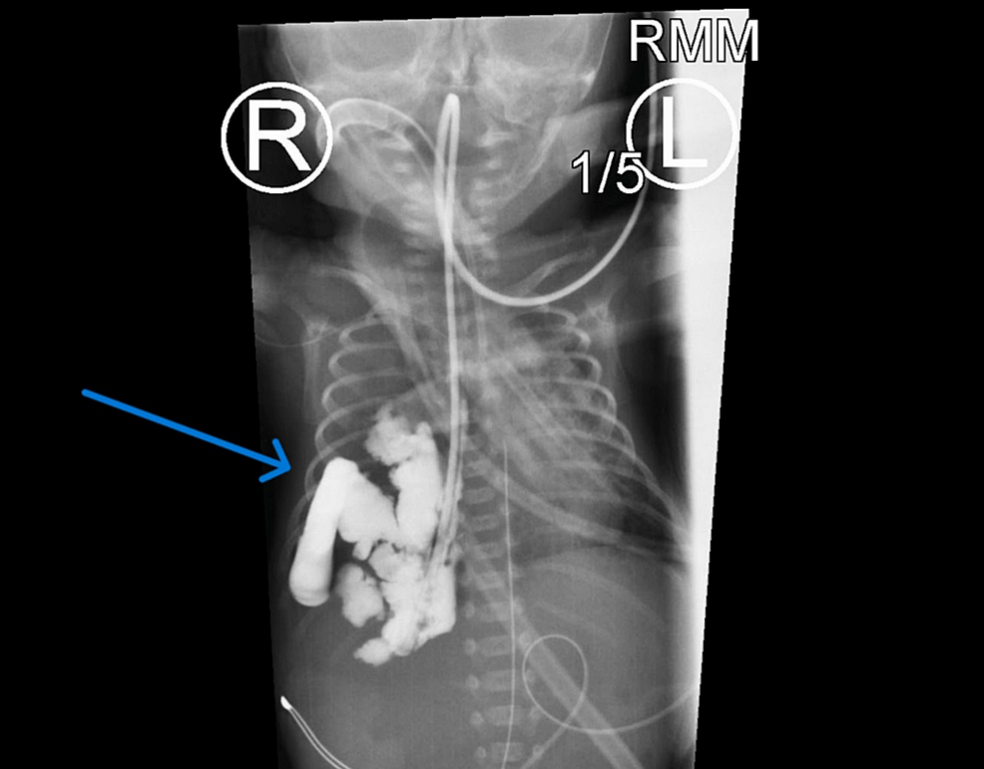
Esophagram with water-soluble contrast depicting what appears to be intestinal contents in the right lung space.

**Figure 3:**
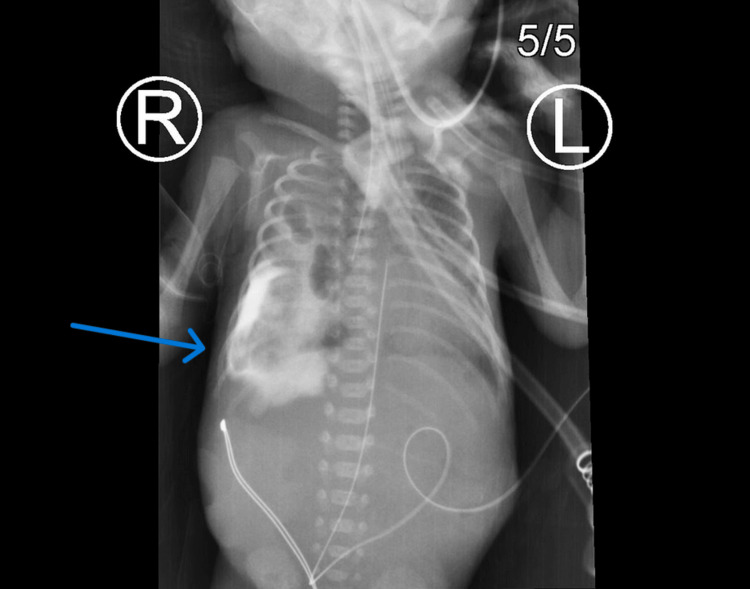
Repeat esophagram three hours later demonstrates even diffusion of the contrast throughout the pleural space.

**Figure 4:**
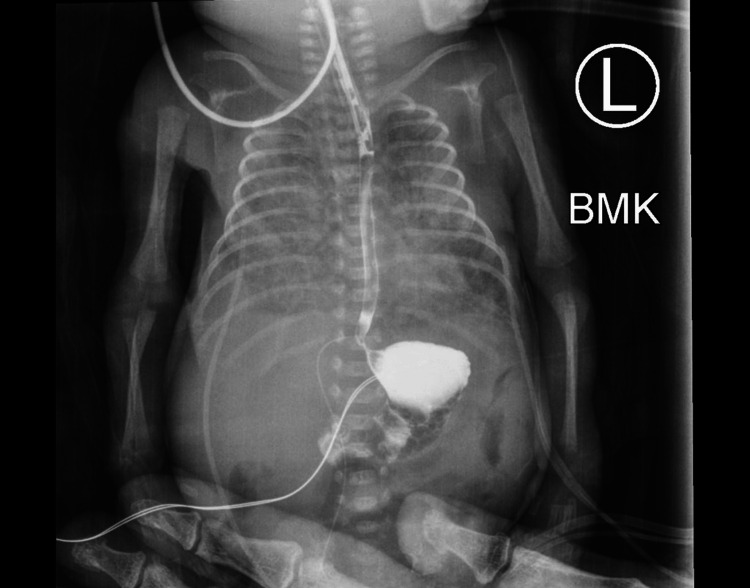
Follow-up esophagram 11 days after initial diagnosis shows normal esophageal and gastric contrast coating. Contrast can be seen coating the length of the esophagus without leakage into the pleural spaces. Normal pooling of the contrast into the stomach can also be observed. An adult hand can be seen at the bottom holding the patient steady for the imaging exam.

Five days later, on October 8, 2022, the clinical team noticed abnormal OGT positioning on a daily chest radiograph (Figure 1) as well as a high pH on an attached pH monitor. This was concerning for an esophageal perforation or abnormal anatomy, although the acuity of the finding was more consistent with perforation. A bedside abdominal ultrasound was performed and showed a normal liver situs, a somewhat distended stomach, and a seemingly normal anatomy of the abdomen without malrotation or gross heterotaxy. An esophagram was then performed using water-soluble Omnipaque contrast (GE HealthCare, Chicago, IL, United States). This exam demonstrated a serpiginous collection of contrast within the right chest resembling a small bowel, leading to the consideration of a congenital diaphragmatic hernia (Figure 2). To exclude a diagnosis of diaphragmatic hernia, a repeat delayed radiograph of the chest demonstrated that the contrast had diffused evenly throughout the right pleural space, establishing the diagnosis of esophageal perforation (Figure 3). Contrast material was drained out of the pleural space, and then the pleural space was lavaged with normal saline at the time of the conclusion of this esophagram. The OGT was removed, and the patient was managed non-surgically with meropenem and flagyl, a 10-day course of total parenteral nutrition, and close observation. Eleven days later, a repeat esophagram on October 19, 2022, demonstrated healing of esophageal perforation with contrast contained in the stomach and esophagus (Figure 4). The physical exam at this time was unremarkable. Trophic feeds via OGT were restarted, and meropenem and flagyl were discontinued.

During these events, this patient was also suffering from neonatal respiratory distress syndrome (NRDS) diagnosed around the time of admission to the NICU, a bilateral intraventricular germinal matrix hemorrhage found on October 5, 2022, and a patent ductus arteriosus (PDA) about 2 mm in size. On October 17, 2022, the patient began to develop head swelling, which grew to involve the neck as well over a period of about one day. No arterial or venous thrombi were found on the imaging workup of this swelling. On October 24, 2022, the swelling of the head and neck acutely worsened. An echocardiogram evaluation of the PDA showed a very mild increase in size to 3 mm in diameter. Two days later, severe acidosis, neutropenia, and widespread body swelling, all consistent with severe sepsis, began to develop. Gradually, this patient developed hypotension with bradycardia, and despite the administration of vasopressors, this patient lost his pulse and was pronounced deceased on October 27, 2022.

## Discussion

Neonatal esophageal perforation is a rare complication of enteric instrumentation. Prematurity and low birth weight are risk factors [1,5]. The thin and underdeveloped esophagus can tear during tube placement and is associated with significant morbidity and mortality [2,3]. The diagnosis is commonly made on post-enteric tube placement radiographs. The enteric tube will have non-esophageal anatomical positioning and can be misidentified as a right main stem bronchus enteric tube placement, esophageal atresia (EA), malrotation, and congenital diaphragmatic hernia [6,7]. Right mainstem bronchus placement was low on this team's differential as the patient's esophagram images did not visually appear consistent with this.

EA is another differential diagnosis that is important to work up in presentations such as this patient. EA is diagnosed in the prenatal period about 33% of the time with ultrasound. Polyhydramnios is a key finding prompting this ultrasonographic exam [8]. Concern for EA was low with this patient, as the patient was passing OGT feeds as early as the first day of life. Malrotation involves the abnormal anatomical positioning of the small intestines due to improper rotation of the bowels during fetal development while the bowels are extruded from the fetal abdomen. The gold-standard test for diagnosing malrotation is the upper gastrointestinal (GI) study, though ultrasound is the common first-line exam due to the ease of performing this exam at the bedside [9]. When performing an ultrasound in this context, the whirlpool sign is commonly looked for in addition. This sign is seen in malrotation due to the superior mesenteric vein (SMV) and mesentery twisting around the superior mesenteric artery (SMA) [10]. An ultrasound exam was performed on this patient and did not find this sign or other signs of malrotation or volvulus. A congenital diaphragmatic hernia (CDH) was a major concern for this patient, despite the rare occurrence of right-sided congenital diaphragmatic hernias. Right-sided CDH only makes up about 15% of all CDH cases, and low birth weight is the chief risk factor as the underdeveloped liver becomes less protective against CDH on the right side [10].

Clinically, the neonate with esophageal perforation can often present, as in this case, stable with no change in vitals or physical exam. However, there may be other signs and symptoms. The trauma of the tube piercing the esophagus may produce hemoptysis. The subsequent air infiltration into the pleural spaces may result in dyspnea and pneumothorax. The air leak from the esophagus may also manifest as subcutaneous emphysema [6].

A water-soluble contrast esophagram can confirm the diagnosis of an esophageal perforation in a neonate [4]. Images from such an esophagram may show diffuse contrast spread throughout the pleura, as demonstrated in Figure 3. If CDH is suspected, such as in this case, esophageal perforation can be distinguished by waiting for contrast diffusion conforming to the pleural space to occur over a period of approximately 30 minutes (Figures 2, 3), as performed in this patient's case. Additionally, the absence of contrast in the stomach and small bowel supports the diagnosis of perforation versus CDH [11]. The resolution of esophageal perforation is also confirmed with a follow-up esophagram, which should demonstrate a coating of the esophagus with contrast without leakage into any nearby structures. Turning the patient over about three times and then repeating imaging with fluoroscopy is helpful during an esophagram exam, as this lets the contrast coat the entirety of the esophagus and stomach [11].

The majority of neonatal esophageal perforations are treated non-operatively. The enteric tube is slowly withdrawn from the esophagus, and the injury is allowed to heal, with most defects closing in 10-14 days. The neonate is kept nil per os (NPO), placed on total parenteral nutrition (TPN), and started on broad-spectrum antibiotics to cover anaerobic and enteric bacteria [12-14]. Once healed, an enteric tube can be inserted up to the cervical esophagus with water-soluble contrast administered for visualization of the esophagus and stomach. If contrast is contained only within the esophagus and stomach, then the perforation may be considered resolved and the enteric tube may be advanced with tube feeds restarted.

## Conclusions

Esophageal perforation is an uncommon, though potentially fatal diagnosis. Esophageal perforation may present somewhat similarly to other diagnoses involving the upper GI tract, such as congenital diaphragmatic hernia, esophageal atresia, and malrotation. Esophagram with water-soluble contrast is the key exam to help differentiate perforation from these diagnoses and is also used to monitor for resolution. An ultrasound exam of the abdomen and chest is also helpful in narrowing the differential. Treatment of esophageal perforation in the neonate is mainly non-operative, consisting primarily of tube withdrawal, NPO diet, and broad-spectrum antibiotics.
